# Some Wilker and Cusa type inequalities for generalized trigonometric and hyperbolic functions

**DOI:** 10.1186/s13660-018-1644-8

**Published:** 2018-03-02

**Authors:** Li-Guo Huang, Li Yin, Yong-Li Wang, Xiu-Li Lin

**Affiliations:** 1 College of Science, Binzhou University, Binzhou, China; 2 College of Mathematics and Systems Science, Shandong University of Science and Technology, Qingdao, China; 3 School of Mathematical Sciences, Qufu Normal University, Qufu, China

**Keywords:** 33B10, Wilker type inequality, Cusa type inequality, Arithmetic–geometric means inequality, Power means inequality, Generalized trigonometric function, Generalized hyperbolic function

## Abstract

The authors obtain some Wilker and Cusa type inequalities for generalized trigonometric and hyperbolic functions and generalize some known inequalities.

## Introduction

It is well known from basic calculus that

1.1 $$ \arcsin x= \int_{0}^{x} \frac{1}{(1-t^{2})^{1/2}}\,dt $$

for $0\leq x\leq1$ and

1.2 $$ \frac{\pi}{2}=\arcsin1= \int_{0}^{1} \frac{1}{(1-t^{2})^{1/2}}\,dt. $$

For $1< p<\infty$ and $0 \leq x \leq1$, the arc sine may be generalized as

1.3 $$ \arcsin_{p} x = \int_{0}^{x} {\frac{1}{(1-t^{p})^{1/p}}}\,dt $$

and

1.4 $$ \frac{\pi_{p}}{2} = \arcsin_{p} 1= \int_{0}^{1} {\frac{1}{ (1-t^{p})^{1/p}}}\,dt. $$

The inverse of $\arcsin_{p}$ on $[0,\frac{\pi_{p}}{2} ]$ is called the generalized sine function, denoted by $\sin_{p}$, and may be extended to $(-\infty, \infty)$. In the same way, we can define the generalized cosine function, the generalized tangent function, and their inverses, and also the corresponding hyperbolic functions. For their definitions and formulas, one may see recent references [1–3].

In [2], some classical inequalities for generalized trigonometric and hyperbolic functions, such as Mitrinović–Adamović inequality, Huygens’ inequality, and Wilker’s inequality, were generalized. In [3], some new second Wilker type inequalities for generalized trigonometric and hyperbolic functions were established. In [4], some Turán type inequalities for generalized trigonometric and hyperbolic functions were presented. Very recently, a conjecture posed in [5] was verified in [1]. For more about the Wilker type inequality and Huygens type inequalities, the reader may see [6–13].

In this paper, we establish some new Wilker and Cusa type inequalities for the generalized trigonometric and hyperbolic functions. Some known inequalities in [3] are the special cases of our results.

## Lemmas

### Lemma 2.1

([3, Lemma 2.7])

*For* $p \in(1,\infty)$, *we have*

2.1 $$ \cos_{p}^{\alpha}x < \frac{\sin_{p} x}{x}< 1,\quad x\in \biggl(0,\frac{\pi _{p}}{2} \biggr) $$

*and*

2.2 $$ \cosh_{p}^{\alpha}x< \frac{\sinh_{p} x}{x}< \cosh_{p}^{\beta}x,\quad x>0, $$

*where the constants* $\alpha=\frac{1}{p+1}$ *and* $\beta=1$ *are the best possible*.

### Lemma 2.2

([3, Theorem 3.5])

*For* $p \in(1,2]$, *then*

2.3 $$ \biggl(\frac{x}{\sin_{p} x} \biggr)^{p} + \frac{x}{\tan_{p} x} > 2,\quad x\in \biggl(0,\frac{\pi_{p}}{2} \biggr). $$

### Lemma 2.3

([14])

*Let* $a>0, b>0$ *and* $r\geq1$, *then*

2.4 $$ (a+b)^{r} \leq2^{r-1} \bigl(a^{r}+b^{r} \bigr). $$

### Lemma 2.4

([15])

*Let* $a_{k}>0, k=1,2,\ldots,n$, *then*

2.5 $$ \frac{a_{1}+a_{2}+\cdots+a_{n}}{n}\geq \sqrt[n]{(1+a_{1}) (1+a_{2})\cdots(1+a_{n})} -1\geq\sqrt[n]{a_{1} a_{2} \cdots a_{n}}. $$

### Lemma 2.5

([2, Theorem 3.4])

*For* $p\in[2,\infty)$ *and* $x\in (0,\frac{\pi_{p}}{2} )$, *then*

2.6 $$ \frac{\sin_{p} x}{x}< \frac{x}{\sinh_{p} x}. $$

### Lemma 2.6

*For* $p\in[2,\infty)$ *and* $x\in (0,\frac{\pi_{p}}{2} )$, *we have*

2.7 $$ \biggl(\frac{\sin_{p} x}{x} \biggr)^{p}< \frac{x}{\sinh_{p} x}. $$

### Proof

Using Lemma 2.5 and $\frac{{\sin_{p} x}}{x} < 1$, we have

2.8 $$ \frac{x}{{\sinh_{p} x}} > \frac{{\sin_{p} x}}{x} > \biggl( { \frac{{\sin _{p} x}}{x}} \biggr)^{p}. $$

This implies inequality (2.7). □

### Lemma 2.7

([2, Corollary 3.10])

*For* $p\in[2,\infty)$ *and* $x\in (0,\frac{\pi_{p}}{2} )$, *then*

2.9 $$ \biggl( \frac{x}{{\sinh_{p} x}} \biggr)^{p+1}< { \frac{{\sin_{p} x}}{x}}. $$

### Lemma 2.8

([2, Theorem 3.22])

*For* $p\in(1,2]$, *the double inequality*

2.10 $$ \frac{\sin_{p} x}{x}< \frac{\cos_{p} x+p}{1+p}\le\frac{\cos_{p} x+2}{3} $$

*holds for all* $x\in (0,\frac{\pi_{p}}{2} ]$.

## Main results

### Theorem 3.1

*For* $x\in (0,\frac{\pi_{p}}{2} )$, $p\in(1,\infty)$, *and* $\alpha-p\beta\leq0$, $\beta>0$, *we have*

3.1 $$ \biggl(\frac{\sin_{p} x}{x} \biggr)^{\alpha}+ \biggl( \frac{\tan_{p} x}{x} \biggr)^{\beta}> 2. $$

### Proof

From the arithmetic geometric means inequality and Lemma 2.1, it follows that

$$\begin{aligned} \biggl(\frac{\sin_{p} x}{x} \biggr)^{\alpha}+ \biggl(\frac{\tan_{p} x}{x} \biggr)^{\beta}&\geq2 \biggl(\frac{\sin_{p} x}{x} \biggr)^{\frac{\alpha}{2}} \biggl(\frac{\tan_{p} x}{x} \biggr)^{\frac{\beta}{2}} \\ & = 2 \biggl(\frac{\sin_{p} x}{x} \biggr)^{\frac{\alpha+\beta}{2}} \biggl(\frac{1}{\cos_{p} x} \biggr)^{\frac{\beta}{2}} \\ & > 2 \biggl(\frac{\sin_{p} x}{x} \biggr)^{\frac{\alpha+\beta}{2}} \biggl(\frac{\sin_{p} x}{x} \biggr)^{-\frac{(p+1)\beta}{2}} \\ &= 2 \biggl(\frac{\sin_{p} x}{x} \biggr)^{\frac{\alpha-p\beta}{2}} \\ &\geq2. \end{aligned}$$

 □

### Remark 3.1

If $p=\alpha=2, \beta=1$, inequality (3.1) turns into

3.2 $$ \biggl(\frac{\sin x}{x} \biggr)^{2}+ \frac{\tan x}{x} > 2. $$

Inequality (3.2) is called the first Wilker inequality in [16].

### Remark 3.2

If $\alpha=2p, \beta=p$, and $p\geq2$, then $\alpha-p\beta=2p-p^{2}\leq0$. So, inequality (3.1) reduces to

3.3 $$ \biggl(\frac{\sin_{p} x}{x} \biggr)^{2p}+ \biggl( \frac{\tan_{p} x}{x} \biggr)^{p} > 2. $$

### Theorem 3.2

*For* $p\in(1,2], x\in (0,\frac{\pi_{p}}{2} )$, *and* $\alpha-p\beta\leq0, \beta\leq-1$, *we have*

3.4 $$ \biggl(\frac{\sin_{p} x}{x} \biggr)^{\alpha}+ \biggl( \frac{\tan_{p} x}{x} \biggr)^{\beta}>2. $$

### Proof

Using $\frac{x}{\sin_{p} x}\geq1$ and $\alpha-p\beta\leq0$, we have

$$\begin{aligned} \biggl(\frac{\sin_{p} x}{x} \biggr)^{\alpha}+ \biggl(\frac{\tan_{p} x}{x} \biggr)^{\beta} &= \biggl(\frac{x}{\sin_{p} x} \biggr)^{-\alpha}+ \biggl(\frac{x}{\tan_{p} x} \biggr)^{-\beta} \\ &= \biggl(\frac{x}{\sin_{p} x} \biggr)^{-p\beta} \biggl(\frac{x}{\sin_{p} x} \biggr)^{p\beta-\alpha}+ \biggl(\frac{x}{\tan_{p} x} \biggr)^{-\beta} \\ &\geq \biggl[ \biggl(\frac{x}{\sin_{p} x} \biggr)^{p} \biggr]^{-\beta}+ \biggl(\frac{x}{\tan_{p} x} \biggr)^{-\beta}. \end{aligned}$$

Applying Lemmas 2.2 and 2.3, we obtain

$$\begin{aligned} \biggl(\frac{\sin_{p} x}{x} \biggr)^{\alpha}+ \biggl(\frac{\tan_{p} x}{x} \biggr)^{\beta} \geq2^{1+\beta} \biggl[ \biggl(\frac{x}{\sin_{p} x} \biggr)^{p}+\frac {x}{\tan_{p} x} \biggr]^{-\beta} >2. \end{aligned}$$

This completes the proof. □

Using the same method as that in Theorem 3.1, we can easily obtain the following Theorem 3.3 by Lemma 2.1 and the arithmetic and geometric means inequality. We omit the proof for the sake of simplicity.

### Theorem 3.3

*For* $p\in(1,\infty), x\in (0,\infty)$, *and* $\alpha-p\beta\leq0, \beta>0$, *then*

3.5 $$ \biggl(\frac{\sinh_{p} x}{x} \biggr)^{\alpha}+ \biggl( \frac{\tanh_{p} x}{x} \biggr)^{\beta}>2. $$

### Remark 3.3

Taking $\alpha=2, \beta=1$ and $p=2$ in inequality (3.5), we have

3.6 $$ \biggl(\frac{\sinh x}{x} \biggr)^{2}+ \frac{\tanh x}{x} > 2, $$

which is the (4) in Theorem 1 of [7]. Inequality (3.6) is called the first hyperbolic Wilker inequality.

### Remark 3.4

Taking $\alpha=2p, \beta=p$, and $p\in[2,\infty)$, we have

3.7 $$ \biggl(\frac{\sinh_{p} x}{x} \biggr)^{2p}+ \biggl( \frac{\tanh_{p} x}{x} \biggr)^{p} > 2. $$

### Theorem 3.4

*For all* $x\in (0,\frac{\pi_{p}}{2} )$ *and* $\alpha-p\beta\leq0, \beta>0$, *we have*

3.8 $$ \biggl[1+ \biggl(\frac{\sin_{p}x}{x} \biggr)^{\alpha}\biggr] \biggl[1+ \biggl(\frac {\tan_{p}x}{x} \biggr)^{\beta}\biggr] >4 $$

*and*

3.9 $$ \biggl(\frac{\sin_{p}x}{x} \biggr)^{\alpha}+ \biggl( \frac{\tan_{p}x}{x} \biggr)^{\beta}>2\sqrt{ \biggl[1+ \biggl( \frac{\sin_{p}x}{x} \biggr)^{\alpha}\biggr] \biggl[1+ \biggl( \frac{\tan_{p}x}{x} \biggr)^{\beta}\biggr]} -2>2. $$

### Proof

Setting $n=2, a_{1}= (\frac{\sin_{p}x}{x} )^{\alpha}$ and $a_{2}= (\frac{\tan_{p}x}{x} )^{\beta}$ in Lemma 2.4, we have

$$\begin{aligned} & \biggl[1+ \biggl(\frac{\sin_{p}x}{x} \biggr)^{\alpha}\biggr] \biggl[1+ \biggl(\frac {\tan_{p}x}{x} \biggr)^{\beta}\biggr] \\ &\quad \geq \biggl[ \biggl(\frac{\sin_{p}x}{x} \biggr)^{\frac{\alpha}{2}} \biggl( \frac{\tan_{p}x}{x} \biggr)^{\frac{\beta}{2}}+1 \biggr]^{2} \\ &\quad > \biggl[ \biggl(\frac{\sin_{p}x}{x} \biggr)^{\frac{\alpha-p\beta }{2}}+1 \biggr]^{2} \\ &\quad > 4. \end{aligned}$$

Then it follows from Lemma 2.1 that

$$ \biggl(\frac{\sin_{p}x}{x} \biggr)^{\alpha}+ \biggl(\frac{\tan_{p}x}{x} \biggr)^{\beta}>2\sqrt{ \biggl[1+ \biggl(\frac{\sin_{p}x}{x} \biggr)^{\alpha}\biggr] \biggl[1+ \biggl(\frac{\tan_{p}x}{x} \biggr)^{\beta}\biggr]}-2>2. $$

 □

### Remark 3.5

If $n=3$ and $a_{1}=a_{2}= (\frac{\sin_{p}x}{x} )^{\alpha}, a_{3}= (\frac{\tan_{p}x}{x} )^{\beta}$ in Lemma 2.4, it can be easily obtained that

3.10 $$ \biggl[1+ \biggl(\frac{\sin_{p}x}{x} \biggr)^{\alpha}\biggr]^{2} \biggl[1+ \biggl(\frac{\tan_{p}x}{x} \biggr)^{\beta}\biggr]>8 $$

and

3.11 $$ 2 \biggl(\frac{\sin_{p}x}{x} \biggr)^{\alpha}+ \biggl( \frac{\tan_{p}x}{x} \biggr)^{\beta}>3 \sqrt[3]{ \biggl[1+ \biggl( \frac{\sin_{p}x}{x} \biggr)^{\alpha}\biggr]^{2} \biggl[1+ \biggl( \frac{\tan_{p}x}{x} \biggr)^{\beta}\biggr]} -3>3, $$

by a similar method to that in Theorem 3.4 when changing the condition $\alpha-p\beta\leq0$ to $2\alpha-p\beta\leq0$.

### Theorem 3.5

*For* $p\in[2,\infty), t>0$, *and* $x\in (0,\frac{\pi_{p}}{2} ]$, *then*

3.12 $$ \biggl( {\frac{x}{{\sin_{p} x}}} \biggr)^{pt} + \biggl( { \frac{x}{{\sinh _{p} x}}} \biggr)^{t} > 2. $$

### Proof

Applying the AGM inequality $a+b\geq2\sqrt{ab} $ and Lemma 2.6 for $a = ( {\frac{x}{{\sin_{p} x}}} )^{pt}$ and $b = ( {\frac {x}{{\sinh_{p} x}}} )^{t}$, we obtain

$$a + b \ge2\sqrt{ \biggl( {\frac{x}{{\sin_{p} x}}} \biggr)^{pt} \biggl( { \frac{x}{{\sinh_{p} x}}} \biggr)^{t} } > 2. $$

The proof is completed. □

### Theorem 3.6

*For* $p\in[2,\infty), t>0$ *and* $x\in (0,\frac{\pi_{p}}{2} ]$, *then*

3.13 $$ (p+1) \biggl( {\frac{x}{{\sin_{p} x}}} \biggr)^{t} + \biggl( {\frac{x}{{\sinh _{p} x}}} \biggr)^{t} > p + 1. $$

### Proof

From the AGM inequality $(n+1)a+b\geq(n+1)\sqrt[n+1]{a^{n}b} $ and Lemma 2.6, for $a = ( {\frac{x}{{\sin_{p} x}}} )^{t}$ and $b = ( {\frac {x}{{\sinh_{p} x}}} )^{t}$, inequality (3.13) follows readily. □

Applying AGM inequality and Lemma 2.7, Theorems 3.7 and 3.8 can be easily obtained by the similar method as before.

### Theorem 3.7

*For* $p\in[2,\infty), t>0$, *and* $x\in (0,\frac{\pi_{p}}{2} ]$, *then*

3.14 $$ \biggl( {\frac{\sinh_{p} x}{{x}}} \biggr)^{(p+1)t} + \biggl( { \frac{\sin _{p} x}{{x}}} \biggr)^{t} > 2. $$

### Theorem 3.8

*For* $p\in[2,\infty), t>0$, *and* $x\in (0,\frac{\pi_{p}}{2} ]$, *then*

3.15 $$ (p+2) \biggl( {\frac{\sinh_{p} x}{{x}}} \biggr)^{t} + \biggl( {\frac{\sin_{p} x}{{x}}} \biggr)^{t} > p + 2. $$

Finally, we give a Cusa type inequality.

### Theorem 3.9

*For* $p\in(1,2]$ *and* $x\in(0,\frac{\pi_{p}}{2}]$, *the function* $f(x) = \frac{{\ln {\frac{{\sin_{p} x}}{x}}}}{{\ln {\frac{{p + \cos_{p} x}}{p+1}} }} $ *is strictly increasing*. *Consequently*, *we have the following inequality*:

3.16 $$ \biggl( {\frac{{p + \cos_{p} x}}{p+1}} \biggr)^{\alpha}< \frac{{\sin_{p} x}}{x} < \biggl( {\frac{{p + \cos_{p} x}}{p+1}} \biggr)^{\beta}$$

*with the best constants* $\alpha = \frac{{\ln {\frac{{2\sin_{p} \frac{{\pi_{p} }}{2}}}{{\pi_{p} }}} }}{{\ln{\frac{{p + \cos_{p} \frac{{\pi_{p} }}{2}}}{p+1}} }} $ *and* $\beta=1$.

### Proof

A simple computation yields

$$\begin{aligned} &f'(x)\ln^{2} {\frac{{p + \cos_{p} x}}{p+1}} \\ &\quad= \frac{{x\cos_{p} x - \sin_{p} x}}{{x\sin_{p} x}}\ln{\frac{{p + \cos_{p} x}}{p+1}} + \frac{{\cos_{p} x\tan_{p}^{p - 1} x}}{{p + \cos_{p} x}}\ln { \frac{{\sin_{p} x}}{x}} \\ &\quad> {\frac{{x\cos_{p} x - \sin_{p} x}}{{x\sin_{p} x}} + \frac{{\cos_{p} x\tan_{p}^{p - 1} x}}{{p + \cos_{p} x}}}\ln{\frac{{\sin_{p} x}}{x}} \\ &\quad= {\frac{{(x\cos_{p} x - \sin_{p} x)(p + \cos_{p} x) + x\sin_{p} x\cos_{p} x\tan_{p}^{p - 1} x}}{{x\sin_{p} x( p+ \cos_{p} x)}}} \ln{\frac{{\sin_{p} x}}{x}} \\ &\quad= \frac{{\ln {\frac{{\sin_{p} x}}{x}} }}{{x\sin_{p} x( p+ \cos_{p} x)}}g(x), \end{aligned}$$

where

$$g(x) = x\cos_{p}^{2} x\sec_{p}^{p} x + px\cos_{p} x - p\sin_{p} x - \sin_{p} x \cos_{p} x. $$

Since

$$g'(x) = \cos_{p} x\tan_{p}^{p - 1} xh(x), $$

where

$$h(x) = 2\sin_{p} x - px - (2 - p)x\sec_{p}^{p - 1} x, $$

with

$$h'(x) = 2\cos_{p} x - p - (2 - p) \sec_{p}^{p - 1} x- (2 - p) (p - 1)x\sec _{p}^{p - 1} x\tan_{p}^{p - 1} x $$

and

$$\begin{aligned} h''(x) ={}& {-} 2\cos_{p} x\tan_{p}^{p - 1} x - 2(2 - p) (p - 1)\sec_{p}^{p - 1} x\tan_{p}^{p - 1} x \\ & {}- (2 - p) (p - 1)^{2} x\sec_{p}^{p - 1} x \tan_{p}^{p - 1} x \bigl(\tan_{p}^{p-1} x + \csc_{p} x\sec_{p}^{p - 1}x\bigr) < 0. \end{aligned}$$

Hence $h'(x)$ is decreasing on $(0,\frac{\pi_{p}}{2})$. It then follows that $h'(x)< h'(0)=0$, which also implies that $h(x)< h(0)=0$. Hence, $g'(x)<0$, which shows that the function $g(x)$ is also decreasing on $(0,\frac{\pi_{p}}{2})$. The inequality $g(x)< g(0)=0$ indicates that $f'(x)>0$. Hence, $f(x)$ is strictly increasing for $x\in(0,\frac{\pi_{p}}{2})$. As a result, we have $f(0)< f(x)\leq f(\frac{\pi_{p}}{2})$.

Using L’Hôspital’s rule, we obtain that

$$\begin{aligned} f\bigl(0^{+} \bigr)&= \mathop{\lim} _{x \to0^{+} } \frac{{\ln {\frac{{\sin_{p} x}}{x}} }}{{\ln {\frac{{p + \cos_{p} x}}{p+1}} }} \\ &= \mathop{\lim} _{x \to0^{+} } - \frac{{x\cos_{p} x - \sin_{p} x}}{{x\sin_{p} x}}\frac{{p + \cos_{p} x}}{{\cos_{p} x\tan_{p}^{p - 1} x}} \\ &= - (p+1)\mathop{\lim} _{x \to0^{+} } \frac{{x\cos_{p} x - \sin_{p} x}}{{x^{p + 1} }} \\ &=1 \end{aligned}$$

and

$$f \biggl(\frac{\pi_{p}}{2} \biggr)=\frac{{\ln {\frac{{2\sin_{p} \frac{{\pi_{p} }}{2}}}{{\pi_{p} }}}}}{{\ln{\frac{{p + \cos_{p} \frac{{\pi_{p} }}{2}}}{p+1}} }}. $$

The proof is completed. □

## A conjecture

### Conjecture 4.1

*For all* $x\in(0,\frac{\pi_{p}}{2}]$ *and* $p\in(1,2]$, *is the function* $\frac{{\ln\frac{x}{{\sin_{p} x}}}}{{\ln\cosh_{p} x}} $ *strictly increasing*?
